# Supplementary material for: Dynamic transcriptomic profiles of zebrafish gills in response to zinc supplementation
Source: BMC Genomics. 2010 Oct 11;11:553. doi: 10.1186/1471-2164-11-553 (PMC3091702; doi:10.1186/1471-2164-11-553)
Supplement: Additional file 2 — Interactive Direct Interaction Network representing the molecular interactions between zinc, copper, iron, calcium and proteins encoded by transcripts changed by zinc supplementation. Mini web-site containing index.html and hyperlinked pages in subdirectory describing a Direct Interaction Network automatically generated based on curated interactions contained within the proprietary PathwayArchitect database. Ovals represent proteins and the circles symbolize metal ions. Objects are coloured by their abundance in zebrafish at the time-point they were significantly different from the control is a scale from -4 fold (dark green) to +4 fold (dark red). Where significant differences were found at more than one time-point, the colour overlay shows expression at the first instance. Dark blue squares denote 'binding', and light blue squares 'expression'; green squares stand for 'regulation', green diamonds for 'metabolism', and green circles for 'promoter binding'. Arrow heads indicate directionality of the interaction where annotated. All nodes and edges can be further interrogated by selecting the relative area of the image. [file 1471-2164-11-553-S2.zip › PathwayArchitect Zn xs DIN/505679.html]

# REGULATION:

|  |  |
| --- | --- |
| Type | REGULATION |
| Effect | Negative |


---

|  |  |
| --- | --- |
| Score | 0 |


---

|  |  |
| --- | --- |
| Reference Count | 7 |


---

|  |  |
| --- | --- |
| Mechanism | Unknown |


---

|  |  |
| --- | --- |
| Reference:0 || Sentence | "The regulation of calcium (Ca(2+)) sparks and transient calcium-sensitive K(+) (K(Ca)) currents by acute changes in sarcoplasmic reticulum (SR) Ca(2+) load ([Ca(2+)](SR)) was investigated in rat cerebral artery smooth muscle cells using laser-scanning confocal microscopy in combination with patch clamp electrophysiology. [Ca(2+)](SR) was elevated by: (i) increasing the activity of the SR Ca(2+)-ATPase with an anti-phospholamban monoclonal antibody, or (ii) blocking Ca(2+) release from the SR with tetracaine, a membrane-permeant, reversible ryanodine-sensitive Ca(2+) release (RyR) channel blocker." |
| PMID | 12356881 |
| Year | 2002 |
| Species | Rat |
| Journal | J Physiol |
| RefScore | 1 |
| Source | PArchNLP |
  |
|  |  |


---

|  |  |
| --- | --- |
 Reference:1 || Sentence | "Attenuated SR function in the heart was associated with reduced CaMK phosphorylation of the SR Ca2+-cycling proteins such as Ca2+-release channel, Ca2+-pump ATPase, and phospholamban, decreased CaMK activity, and depressed levels of SR Ca2+-cycling proteins." |
| PMID | 12054530 |
| Year | 2002 |
| Species | Rat |
| Journal | Biochem Biophys Res Commun |
| RefScore | 1 |
| Source | PArchNLP |
  ||  |  |


---

|  |  |
| --- | --- |
 Reference:2 || Sentence | "Normalization of Ca(2+) cycling by ablation or inhibition of the SR inhibitor phospholamban (PLN) has prevented cardiac failure in experimental dilated cardiomyopathy and is a promising therapeutic approach for human heart failure." |
| PMID | 12639992 |
| Year | 2003 |
| Species | Mouse |
| Journal | J Clin Invest |
| RefScore | 0 |
| Source | PArchNLP |
  ||  |  |


---

|  |  |
| --- | --- |
 Reference:3 || Sentence | "The antibody and phospholamban phosphorylation appear to release the inhibitory action of phospholamban on Ca2+ pump ATPase, resulting in the stimulation of Ca2+ pump." |
| PMID | 1666413 |
| Year | 1991 |
| Journal | J Mol Cell Cardiol |
| RefScore | 1 |
| Source | PArchNLP |
  ||  |  |


---

|  |  |
| --- | --- |
 Reference:4 || Sentence | "Dephosphorylated phospholamban inhibits the Ca2+-pump and depresses contractility, whereas phosphorylation of phospholamban by cAMP-activated mechanisms relieves this inhibition and increases contractility." |
| PMID | 9799653 |
| Year | 1998 |
| Species | Mouse |
| Journal | J Mol Cell Cardiol |
| RefScore | 0 |
| Source | PArchNLP |
  ||  |  |


---

|  |  |
| --- | --- |
 Reference:5 || Sentence | "Dephosphorylated PLB suppresses the SR Ca(2+) pump activity, whereas phosphorylation of PLB leads to deinhibition." |
| PMID | 11852250 |
| Year | 2002 |
| Species | Rat |
| Journal | Trends Cardiovasc Med |
| RefScore | 1 |
| Source | PArchNLP |
  ||  |  |


---

|  |  |
| --- | --- |
 Reference:6 || Sentence | "The regulatory protein phospholamban exerts a physiological inhibitory effect on the sarcoplasmic reticulum (SR) Ca2+ pump that is relieved with phosphorylation." |
| PMID | 9612393 |
| Year | 1998 |
| Species | Mouse |
| Journal | Am J Physiol |
| RefScore | 0 |
| Source | PArchNLP |
  |


---

|  |  |
| --- | --- |
